# Artificial intelligence in pathologic myopia: a review of clinical research studies

**DOI:** 10.3389/fmed.2025.1572750

**Published:** 2025-04-23

**Authors:** Xinying He, Yun Wang, Xiaojun Zhang, Wei Chi, Weihua Yang

**Affiliations:** ^1^The Second Affiliated Hospital of Nanjing Medical University, Nanjing, Jiangsu, China; ^2^Shenzhen Eye Hospital, Shenzhen Eye Medical Center, Southern Medical University, Shenzhen, Guangdong, China

**Keywords:** artificial intelligence, pathologic myopia, diagnosis, classification, prediction

## Abstract

Myopia is a significant global health challenge, with the incidence of pathologic myopia (PM) on the rise. PM-related fundus diseases have become a leading cause of irreversible blindness. Early detection and treatment are crucial for the prevention and control of myopia. Recent advancements in artificial intelligence (AI), particularly in machine learning and deep learning algorithms, have shown promising results in the field of PM in ophthalmology. This review explores the latest developments in AI technology for managing PM, emphasizing its role in screening and diagnosis, grading and classification, and predictive assessment. AI has shown significant potential for clinical application in PM management, enhancing its intelligent, precise, and efficient practices.

## 1 Introduction

Myopia, a prevalent global condition, is characterized by focused light in front of the retina due to the eye's extended axial length (AL) or changes in the refractive medium (e.g., cornea and lens) or abnormal accommodation of the eye, leading to blurred vision (1). Myopia can progress to high myopia (HM) and pathologic myopia (PM). HM is defined as a significant lengthening of the AL of the eye with a refractive error usually at or above −6.00 D. Typically, the AL of the eye is more than 26.5 mm. In comparison, PM is defined as structural changes in the eye due to excessive elongation of AL, resulting in severe visual impairment and complications such as posterior scleral staphyloma, lacquer cracks, choroidal neovascularization, macular hemorrhage, and myopic maculopathy (MM). PM emerges with characteristic fundus lesions, indicating a more advanced stage compared to HM, which highlights a significantly higher disease-related risk (2). With advances in ophthalmic technology, significant breakthroughs have been achieved in the diagnosis and treatment of PM. Advanced imaging tools such as color fundus photography (CFP) and optical coherence tomography (OCT) have revealed new pathologic features such as leopard-shaped fundus and myopia-associated MM. At the same time, new treatments for these lesions and their complications have shown remarkable results. For example, anti-VEGF therapy has been shown to be effective in treating macular neovascularization triggered by PM, while vitreoretinal surgery has emerged as an effective means of managing traction macular detachment. Posterior scleral reinforcement has been proposed as a treatment option to address the problem of increasing axial growth, and its effectiveness and limitations have been clinically.

The prevalence of myopia, particularly in East and Southeast Asia, presents a major health challenge. Predictions suggest a significant rise in HM by 2050, with the prevalence of myopia in the world population from 22.9% to 49.8% (3–5). The widespread occurrence of myopia (30%−50%), especially PM (1%−3%), underlines the urgent need for effective management strategies to reduce its heavy societal impact (6). Early detection and regular monitoring are essential for managing PM, emphasizing the gap between growing diagnostic needs and limited medical resources, posing a significant public health challenge.

In the realm of managing such challenges, AI emerges as a pivotal tool, encompassing machine learning (ML) and deep learning (DL) which employ deep neural networks (DNNs) to solve complex problems. Convolutional neural networks (CNNs), a specific type of DNN, are adept at processing image data, making them particularly useful in ophthalmology. The capability of AI to analyze vast amounts of data offers unprecedented support in diagnosing, grading, and predicting ocular diseases (7). Its application in PM, through accurate analysis and interpretation of fundus images, marks a significant advancement in ophthalmology, potentially revolutionizing PM management (8–13). The China Alliance of Research in High Myopia (CHARM), a large-scale research project planned to cover more than 100,000 Chinese patients, aims to investigate the diagnosis, progression, and genetic factors of HM (14). AI applications for myopia have now been identified and synthesized by many researchers, but there is not a complete narrative of AI applications for PM. Complications of PM bring serious health hazards and economic burdens to patients and society, so it is urgent to fully recognize the application of AI in PM.

This review delves into the application of AI technology in PM, highlighting significant research breakthroughs and their value in clinical practice. AI is transforming PM management through a stepwise approach:

1. Screening and Diagnosis: AI-powered image analysis automates early detection of PM lesions, significantly improving diagnostic sensitivity compared to manual review.
2. Grading and Classification: Building upon diagnostic findings, AI further quantifies lesion features to objectively classify disease stages.
3. Predictive Assessment: By integrating longitudinal data and lesion characteristics, AI models generate personalized progression risks, enabling proactive interventions.

**Figure 1:**
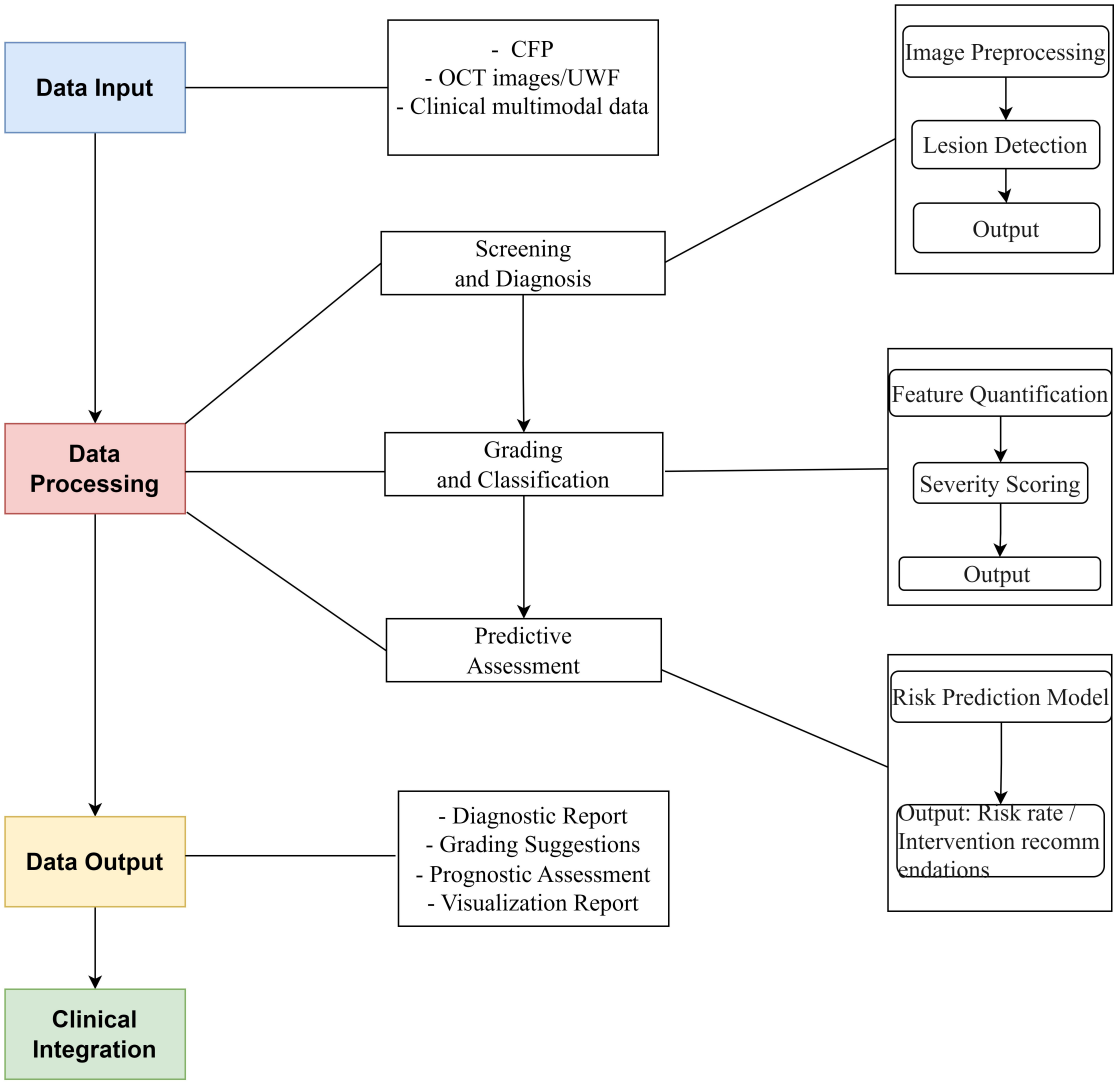
AI pipeline for PM clinical application.

This pipeline (Figure 1)—from detection to stratification to prognosis—demonstrates AI's capability to address the full clinical spectrum of PM.

## 2 Retrieval methods

On 31 January 2025, we conducted a thorough search of the PubMed database and WOS Core Collection. We reviewed the latest research on AI in the field of PM. Our focus was on screening and diagnostic methods, classification and grading systems, and prediction models for disease progression. We used search terms including “artificial intelligence”, “machine learning”, “deep learning”, “ensemble learning”, “reinforcement learning”, “transfer network”, “neural network”, “supervised learning”, “computer vision system”, “computational intelligence”, “evolutionary computation”, “large language model”, “pathologic myopia”, “high myopia”, “myopic maculopathy”, and “progressive myopia”.

Inclusion criteria: The inclusion criteria were as follows:

1. AI technology application: Research studies that utilize AI technology for the diagnosis, grading, and prognostic evaluation of PM.
2. Detailed model development: Research studies that provide a comprehensive description of the AI model construction, including the training and validation processes.

Exclusion criteria: The exclusion criteria were as follows:

1. Non-human research studies: Research studies that involve animal models or *in vitro* experiments rather than human subjects.
2. Review, conference papers, and non-peer-reviewed articles: Research studies that are review articles, conference papers, or articles that have not undergone peer review.
3. Clinical research articles unrelated to AI or PM.

**Figure 2:**
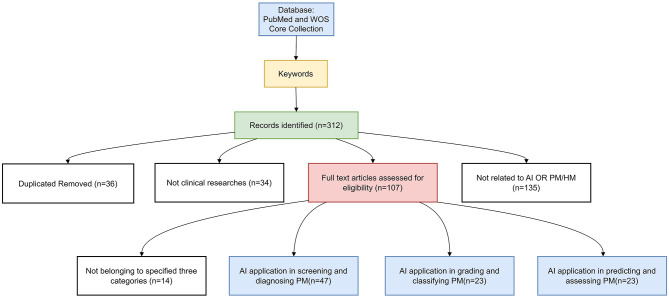
Illustration of the literature search strategy in PUBMED and WOS core collection.

After collecting the relevant literature, we manually screened the titles and abstracts to match the research, and the screening process details are provided in Figure 2.

Analyzing the latest AI research findings in the field of PM and categorizing them based on their content, the application of AI in managing PM is prominently showcased in three primary aspects: screening and diagnosis, grading and classification, and predictive modeling for assessing PM progression.

## 3 Applications of AI in screening and diagnosis of PM

Early identification and accurate diagnosis of PM are crucial for the effective prevention and management of its complications (15). Table 1 lists AI applications in the diagnosis of PM (Literature not listed is not clinical research studies in multiple case centers and could not be included in the table).

**Table 1:** The application of AI in the screening and diagnosis of PM/HM.

**Authors**	**Modalities**	**Sample size**	**Algorithms**	**AUC (%)**	**Accuracy (%)**	**Sensitivity (%)**	**Specificity (%)**
Zhu et al. (16)	CFP	2,400	DL	>95.0	95.6	96.1	99.6
Gu et al. (18)	CFP	4,795	DL	–	93	85	94
Rauf et al. (20)	CFP	–	CNN	98.5	–	–	–
Ren et al. (22)	CFP	1,156	DL	–	–	98.17	93.06
Wang et al. (25)	CFP	7,606	CNN/TL	95.0–100.0	93.2–99.8	90.8–96.8	93.3–99.9
Tan et al. (26)	CFP	226,686	CNN	97.3	–	95	86.9
Lu et al. (28)	CFP	17,330	DL	99.3	97.7	97.7	97.2
Qian et al. (29)	CFP	4,603	DL	–	–	80.1	94.6
Li et al. (30)	CFP	36,515	DCNN	97.0–99.8	93.0–96.9	90.8–93.3	98.7–99.6
Zhang et al. (31)	CFP	1,391	DL	99.9	96.8	83.1	95.6
Choi et al. (32)	OCT	690	CNN	99	100	–	–
Ye et al. (33)	OCT	2,342	DL	92.7–97.4	–	–	–
Li et al. (34)	OCT	5,505	CNN	96.1–99.9	–	90.0–100.0	90.5–96.5
Li et al. (39)	OCT	720	DL	–	–	92.4	99.8
Xu et al. (40)	OCT	800	DL	99.5	–	–	–
Zhang et al. (27)	UWF	2,644	DCNN	–	93	90.7	95.2
Mao et al. (46)	UWF	317	DL/TL	–	98.2	71.4	99.4

### 3.1 AI screening and diagnosis of fundus images for PM

AI plays a pivotal role in enhancing the capabilities of primary care providers in detecting and managing PM by automating the screening process and facilitating timely referrals. This advancement is further supported by the development of imaging technologies ranging from CFP and OCT to ultra-widefield (UWF) imaging (16), which has dramatically improved the understanding of myopic fundus lesions (17). The target population for this task is at-risk individuals in the general population, especially adolescents and children, as they are at a critical period in the development of myopia.

#### 3.1.1 Color fundus photography

Research initiatives have successfully used various AI models (e.g., EfficientNet) based on CFP to diagnose common retinal diseases such as PM, glaucoma, and diabetic retinopathy with high accuracy (18–21). The multi-disease diagnostic tool PADAr has been developed for primary eye screening, demonstrating the potential of AI to increase the rate of early diagnosis of PM in the primary care setting (22).

Developing an automatic, accurate, and non-invasive screening system for PM patients through AI can significantly conserve time and resources for both clinicians and patients. CNNs, specialized in processing lattice-structured data, have demonstrated superior performance in the automatic screening and diagnosis of PM. The pre-processed fundus photographs were feature extracted by the CNN model and categorized into normal fundus images and pathologic myopic fundus, achieving the best AUC of 0.9845 (23). However, the problem of overfitting cannot be avoided, and data augmentation mode was matched to the image dataset, achieving an accuracy increase of 2.85% (24). DL models, such as the PM-AI system (25) and MyopiaDETR (26), not only effectively detect the location of lesions but can also suggest the presence of myopic choroidal neovascularization (mCNV) (27–30). MM is a series of macular lesions associated with PM, characterized by macular atrophy, lacquer cracks, choroidal neovascularization, and posterior staphyloma. DL models show great potential in screening the diagnosis of MM (31–33). Furthermore, self-supervised learning is integrated with DL to make a more precise diagnosis (34).

#### 3.1.2 Optical coherence tomography and ultra-widefield images

Fundus imaging presents significant challenges for PM. The evolution of modalities for ophthalmic imaging provides a solid foundation for entering the era of healthcare intelligence. Choi et al. (35) comparatively assessed the ability of three DL models to screen for HM using OCT imaging, with ResNet 50 performing the best. Innovations such as the application of models like Inception ResnetV2 and ResNeSt101 (36) for detecting fundus complications (retinoschisis, macular hole, retinal detachment, and mCNV) affirm the value of CNNs in achieving high diagnostic accuracy (37). Anatomically, Ota-Itadani et al. (38) used DL on 3D OCT imaging to detect and classify lamina cribrosa defects, assisting in early screening for PM.

The advent of UWF technology has also solved many of the challenges of PM imaging (30). UWF OCT improves the imaging of mCNV, the vitreoretinal interface, and the progression of myopic traction maculopathy (17).

These advancements underscore the transformative potential of AI in the management of PM, highlighting AI's role in refining diagnostic methodologies through enhanced image analysis and the integration of novel technologies.

### 3.2 AI applications of imaging markers and multimodal data analysis for PM

The applications of AI in employing imaging markers and bioinformation for the diagnosis of PM have shown significant advancements. Choroidal thickness and the degree of peripapillary atrophy (PPA) at the optic disc are recognized as effective indicators for assessing PM-related lesions (39–41).

Utilizing AI for precise segmentation and data collection on choroidal characteristics greatly aids in disease diagnosis. Recent research using DL algorithms has demonstrated a high degree of accuracy in distinguishing between the choroidal thickness measured automatically and manually, highlighting AI's capability in achieving detailed analysis with high agreement levels (42–44). Research studies utilizing mask region CNN models have automated the process of segmenting the choroidal region in OCT images of myopic patients, enabling the assessment of myopia severity based on choroidal thickness (45). Innovations such as the Boundary Enhancement Module and the utilization of parallel branches in AI architecture have aimed to improve segmentation accuracy by enhancing boundary features from varied perspectives (46). DL has also facilitated the automatic quantification of the vascular structure of the choroid, allowing the extraction of valuable metrics such as choroidal thickness, area, volume index, and vessel density through automated segmentation (47, 48), while thinner choroidal thicknesses tend to be associated with elongation of AL over 2 years (49). Furthermore, DL models have been employed successfully for noise reduction in OCT-scanned images, achieving the automated three-dimensional reconstruction and segmentation of intrachoroidal cavitation (50). AI applications extend to enhance the accuracy of segmenting high myopic choroid, with U-Net-based sequence models showing promising results (51). Although UWF imaging is primarily used to capture images of the peripheral retina, its 200° panoramic field of view and high resolution also make it possible to display details of the posterior pole clearly. The analysis of UWF imaging has revealed an association between the severity of mCNV and increased retinal blood vessel density and branching, highlighting AI's potential to provide foundational data for PM screening and diagnosis (52). Moreover, AI-measured lacquer crack extension is proved to be related to the progression of mCNV that a larger initial LC area or a faster rate of LC expansion may predict earlier CNV onset (53).

Advancements in AI have enhanced the segmentation accuracy of the optic disc and PPA (54). Measurement of vascular parameters and PPA area with a DL model confirms pathologic mechanisms in the development of PM (55). Through the integration of vision transformer and CNNs in model development, modern algorithms, based on UWF imaging, significantly outperform classical models in segmenting the optic disc and PPA (56). Its ability to handle complex backgrounds and fuzzy boundaries, multi-scale feature extraction, feature fusion, and boundary information processing enable transformers to more accurately segment PPA, fundamentally aiding ophthalmologists in diagnosing and managing conditions related to PM. However, incorporating transformers typically requires larger training datasets, so a lightweight image segmentation network named simple CNN-UNet is proposed for precise segmentation of the optic disc (57). In response to the limitations of retinal atrophy such as unclear boundaries and irregular shapes, ARA-Net-segmented retina has been proposed to detect retinal atrophy in various area sizes (58).

AI significantly enhances the diagnosis of PM by integrating more biological data alongside the direct screening and identification of distinctive fundus images. Zhang et al. (59) developed the PM-BMII, a computer-aided diagnostic framework that smartly merges diverse biomedical data from various sources, such as genetic, demographic, clinical information, and imaging data, improving the diagnostic accuracy of ~4.2–46.3% of PM. Furthermore, AI's capability to discern correlations between multiple potential factors and HM enriches our understanding, as evidenced by Zhang et al.'s (60) discovery of a significant link between increased serum vitamin A levels and HM prevalence. The role of AI extends beyond diagnostic advancement to enhancing healthcare service quality and the efficacy of medical interventions. A comparative research by Fang et al. (61) highlighted the superior educational effectiveness of an AI-driven PM auto-recognition system over traditional teaching methods in improving students' diagnostic accuracy and learning efficiency, showcasing the impact of AI in medical education.

The deployment of AI in the diagnosis of PM presents multiple advantages, such as convenience, speed, and medical resource conservation, alongside ensuring accuracy and enhancing management efficiency. Its automated screening and diagnostic capabilities play a pivotal role in early detection, raising awareness, and mitigating complications. This not only improves the ophthalmologist's ability to deliver timely and personalized interventions but also improves the overall patient experience through more accurate diagnostic results. Consequently, AI's contributions are vital in preserving visual health and alleviating societal burdens.

## 4 Applications of AI in grading and classification of PM

The application of AI in grading and classifying PM is indispensable for determining the severity of damage, the risk of complications, and its impact on visual acuity. This categorization is essential to develop specific treatments, ranging from monitoring the progression in mild cases to preventing complications in severe conditions. The target population for this task is patients who have been diagnosed with PM, which facilitates clinicians to regularly track the progress of PM, assess the effectiveness of treatment, and make timely adjustments to the treatment plan. Table 2 lists AI applications in the grading and classification of PM.

**Table 2:** The application of AI in the grading and classifying of PM/HM.

**Authors**	**Modalities**	**Sample size**	**Algorithms**	**AUC (%)**	**Accuracy (%)**	**Sensitivity (%)**	**Specificity (%)**	**MM grading methods**
Wan et al. (55)	CFP	758	DCNN	99.6–99.7	98.2	–	–	–
Huang et al. (56)	OCT	2,837	CNN	96.1–99.8	–	91.7–97.8	98.3–99.1	–
Hemelings et al. (57)	CFP	1,200	CNN	98.6	–	–	–	–
Lu et al. (28)	CFP	16,428	DL	99.3	97.7	97.7	97.2	–
Du et al. (58)	CFP	7,020	DL	88.1–98.2	92.1	37.8–87.2	94.5–98.3	–
Zheng et al. (59)	CFP	4,642	DL	96.0–99.2	83.6	64.7–96.9	–	–
Sogawa et al. (60)	OCT	910	CNN	97.0–100.0	67.6–96.5	90.6–100.0	94.2–100.0	–
Lu et al. (65)	CFP	32,010	DL	99.5	97.4	93.9	98.2	META-PM
Wu et al. (66)	OCT	1,853	DL	89.5–96.9	85.3–94.2	–	–	ATN
He et al. (67)	OCT	3,945	DL/TL	98.6	96	–	–	ATN
Wan et al. (69)	CFP	1,750	DL	96.6	–	92.3–100.0	98.1–100.0	
Tang et al. (71)	CFP	1,395	DL	99.8	–	–	–	META-PM
Zhu et al. (70)	CFP	4,252	ALFA-Mix+	–	89.6	86.4	97.2	META-PM
Zhang et al. (80)	CFP	2,159	DL	99.5	95.4	95.4	98.9	META-PM

*ALFA-Mix*+*: an active learning algorithm based ALFA-Mix*.

### 4.1 General advances in AI for grading and classification of PM

In general, many advances have been made by many researchers in AI for PM grading and classification. Wan et al. (62) have successfully utilized a DL model for intelligent risk grading of HM in fundus images. The model's ability to automatically classify fundus images into normal fundus, low-risk HM, and high-risk HM categories demonstrates its superiority over traditional assessments by ophthalmologists, with sensitivity and specificity close to 100%. In another research, Huang et al. (63) employed the ResNet-34 architecture to enhance the grading of traction retinopathy in highly myopic eyes across five categories, achieving significant accuracy in identifying varying severities of traction retinopathy. To optimize current automated diagnostic tools, Wang et al. (18) applied lightweight classification models such as MobileNetV3 and ShufflenetV2, which offer the benefits of shorter diagnosis times along with reduced storage and parameter requirements. Hemelings et al. (64) combined PM classification with lesion segmentation for PM complications using 2D fundus images and the transformer architecture, leading to a reduced false-positive rate for PM cases. Furthermore, the recent contributions of Lu (31) and Du et al. (65) in employing the International Classification System (META-PM) for PM identification and classification have been notable. While Du R's research is primarily aimed at predicting the risk of developing HM in children, Lu L's research focused more on identifying and classifying PM, with a particular emphasis on pathologic retinal changes.

### 4.2 Advances in AI for grading and classifying MM

Advances in AI have significantly improved the grading and classifying of MM, a major complication of PM that substantially affects vision quality. MM refers to characteristic degenerative changes in the macula, including chorioretinal atrophy, Fuchs spot, lacquer cracks, posterior staphyloma, and optic disc changes. A CNN model was designed for automatic extraction and quantification of features of the posterior scleral wall from OCT images, which were finally presented as 3D-MRI images (66). The classification of MM is crucial, given its implications on treatment and prognosis. Zheng et al. (67) utilized the EfficientNet model applied to fundus photographs of MM, achieving notable sensitivity and specificity. By exploiting SS-OCT imaging, Sogawa et al. (68) found that DL models could precisely diagnose MM, distinguishing it from non-degenerate conditions. In addition, Du et al. (69) investigated novel imaging biomarkers within the optic disc region of fundus images through ML and radiomics, uncovering features that effectively differentiate between varying degrees of MM severity. Tessellated fundus (TF), well-defined choroidal vessels at the posterior fundus pole, is associated with the development and progression of MM. The choroidal area per unit area exposed on fundus photographs can be used as an assessment metric for deep learning models, reflecting the severity of MM (70–73). Quantified FT was found to migrate toward the macula as myopia progressed by DL techniques, which has potential applications for predicting HM and its complications.

Currently, the META-PM and ATN (A—atrophy; T—traction; N—neovascularization) grading systems are the primary methods for MM assessment. Lu et al. (74) utilized these systems to train DL algorithms for grading retinal fundus images, achieving an accuracy comparable to that of expert evaluations. Based on the META-PM system, DL models were automatically extracted from fundus images of diffuse chorioretinal atrophy (DCA) regions from fundus images, which were quantitatively graded into four classes based on size, density, and ocular biology (75). It has been shown that the presence of DCA and its association with visual function impairment is linked to a reduction in choroidal perfusion and stromal composition (76). However, challenges remain, such as the algorithm's inability to detect posterior staphyloma or accurately diagnose “PLUS” lesions. Wu et al. (77) investigated myopic macular lesions using the ATN system, employing CNNs (ResNet) and MB-ASPP techniques for finer subgroup classifications. This DL approach demonstrated high accuracy, as reflected in the AUC values for A, T, and N classifications. He et al. (78) employed macular OCT images from the ATN system, developing two algorithms, where the latter, utilizing transfer learning, showed improved performance. A gated attention mechanism network (GAMNet) achieves 93.3% accuracy in classifying myopic tractional macular degeneration (79). Ye et al. (36), adopting the ResNet 501 architecture across five distinct models, achieved differentiation of various MM features such as macular choroidal atrophy and myopic tractional maculopathy among others. The ensemble model made by combining different deep learning models performs better on MM classification (80). Comparative research studies of AI models by Wan et al. (81), Zhu et al. (82), and Tang et al. (83) showcased advancements in MM detection and classification, employing techniques such as VOLO-D2 models and the DeepLabv3+ network for lesion segmentation and classification. On the basis of the above META-PM grading, Meng et al. (84) proposed to grade HM by combining the visual index of contrast sensitivity function (CSF) and fundus features, emphasizing the non-negligible role of functional indexes, which help to identify the hidden visual damage in early stage (84). These research studies have enhanced our understanding of myopic retinopathy onset signs and provided innovative approaches for monitoring myopia progression.

## 5 Applications of AI in predicting and assessing PM

The application of AI in predicting and assessing PM is essential for identifying individuals at the highest risk and accurately forecasting future trends early in the disease (Table 3). AI's capability in DL-based classification has demonstrated considerable potential in ophthalmic research for the early prediction and assessment of PM.

**Table 3:** The application of AI in the predicting of PM/HM.

**Authors**	**Modalities**	**Sample size**	**Algorithms**	**AUC (%)**	**Accuracy (%)**	**Projected content**
Zhao et al. (72)	Refraction records	88,250	–	96.5–99.7	–	Spherical equivalent (SE)/high myopia onset
Yoo et al. (73)	OCT	936	DL	81.3	71.4	Uncorrected refractive error
Wang et al. (74)	Clinical and imaging information	967	–	87	–	Visual acuity
Chen et al. (75)	Clinical and imaging information	660	–	85–89	–	Myopic maculopathy Risk
Lu et al. (76)	OCT	710	ML	93.4–95.6	84.7–94.4	Axial length
Li et al. (77)	blood tests	20,870	ML	79	72	Occurrence of retinal detachment
Lin et al. (78)	Refraction records	132,457	RF	80.2–88.8	–	Myopia development
Foo et al. (79)	CFP	9,456	DL	90.0–99.0	–	The 5-year risk of high myopia
Li et al. (80)	Medical records	612 530	ML	99	–	Myopic progression
Guan et al. (81)	Medical records	1,285,609	RF	95.7	97.1	High myopia onset

### 5.1 AI prediction for risk assessment of PM

In the realm of risk assessment for PM, AI methods have been deployed to gauge prospective risk and predict the tendency toward progressive myopia in patients with HM. Zhao et al. (85) employed random forest and gradient boosting decision tree (GBDT) models to predict refraction and the likelihood of developing HM using a refractive data span of 15 years. This approach aids in the early identification and prevention of myopia. Yoo et al. (86) extracted structural features from the posterior segment of OCT images and utilized ML to predict uncorrected refraction, which reduces the risk of overlooking problems associated with PM or HM. Similarly, machine learning models for predicting long-term visual acuity in patients with high myopia have also achieved good results using fundus imaging information (87). In addition, machine learning based on SS-OCT can make predictions about the progression of MM after 10 years based on relevant predictors such as longer AL or thinner choroidal thickness (88). Although AL and choroidal thickness are important predictors of PM, they have no discriminative ability sufficient for prediction. Kim et al. (89) utilized a support vector machine (SVM) to accurately measure the relative tomographic elevation of posterior sclera on OCT images, increasing accuracy by 10% compared to that which only measured AL and choroidal thickness. Lu et al. (90) developed classifiers to predict ocular AL using OCT scanning, while Li et al. (91) continuously monitor and predict retinal detachment in patients with HM using the conditional probability algorithm based on blood parameters designed, with an AUC of ~0.96.AI prediction for myopia risk in visually developing children.

Regarding the assessment of myopia risk in visually developing children, it is crucial to predict refractive changes during this critical period to manage the prevalence of HM and PM adequately. Multiple machine learning algorithms were constructed to confirm the correlation between BMI and HM with an AUC of 85%, with implications for the etiologic progression of HM (92). Lin et al. (93) used random forest on large datasets, alongside factors such as age and spherical lenses, to forecast HM incidence over 8 years. Foo et al. (94) combined DenseNet-121, a deep neural network model, with random forest to evaluate fundus images and clinical data, assessing the likelihood of children developing HM within 5 years. Li et al. (95) aggregated data from various cohorts to predict myopia progression and the risk of HM using ML, achieving an AUC of 0.99. Guan et al. (96) explored risk factors for HM through ML, with random forest showing notable accuracy. Clark et al. (97) introduced “polygenic scoring” (PGS) to predict myopia in children by assessing genetic susceptibility through DL and examining refractive error across different European populations. Ota-Itadani et al. and Liu et al. have further illustrated the potential of AI in PM research, focusing on structural abnormalities in PM patients. Liu et al. (98) and Kang et al. (99) observed optic disc and peripapillary atrophy (PPA) changes related to refractive errors in school-aged children, suggesting the PPA to optic disc area ratio as an early indicator of myopia progression. Tiny retinal vascular changes in children with HM can also be recognized by AI, such as a decrease in arterial branching angle and vascular arteriovenous ratio, which can help identify trends in HM earlier (100, 101).

### 5.2 AI prediction for treatment outcomes in PM

AI has demonstrated its potential to effectively predict treatment outcomes and efficacy in patients with PM. For instance, Zhou et al. (102) applied AI algorithms to calculate lens power during intraocular lens (IOL) implantation in highly myopic eyes, achieving high prediction accuracy. Notably, AI models such as XGBoost, Hill-RBF, and Kane excelled in cases with an AL exceeding 26 mm. In another research, Guo et al. (103) utilized extreme gradient boosting and support vector regression algorithms to formulate the Zhu-Lu formula, a novel ML approach for predicting post-surgical lens dioptric power in highly myopic patients. Wei et al. (104) developed and compared five ML algorithms based on OCT scans, all of which showed high accuracy and stability in predicting the optimal corrected visual acuity after cataract surgery in highly myopic patients. Yang et al. (105) and Kang et al. utilized a deep neural network to predict visual acuity in patients with PM and mCNV treated with anti-VEGF, achieving an accuracy rate of over 80%. Sawai et al. (106) investigated the impact of the denoising process on the prediction of PM using single-frame optical coherence tomography angiography (OCTA) imaging, highlighting its role in providing fast and high-quality image analysis. Despite these advancements, the AI-based prediction of PM progression remains in its early stages. To enhance the management of PM and improve patient outcomes, it is crucial to increase scientific research investment, foster interdisciplinary collaboration, and conduct more rigorous clinical trials.

## 6 Limitations of AI in PM

While AI technology has made significant strides in various fields, its application in the clinical research and treatment of PM faces notable challenges. One such challenge is the poor quality of retinal images, such as artifacts caused by eye movements during imaging, which impede the accurate identification and diagnosis of ocular microscopic lesions. To address this, Cheng et al. (107) proposed the structure-preserving guided retinal image filtering (SGRIF) method, which enhances image quality by simulating the natural attenuation and scattering of the human eye lens, thereby improving contrast and aiding in the automated diagnosis of ophthalmic diseases such as optic disc analysis. Improving image quality in clinical work is the basis for AI in ophthalmology clinical work.

In addition, several overarching issues persist in AI research and application for PM. First, the issue of data heterogeneity deficits and model generalization bottlenecks represents the foundational barrier to advancing AI applications in PM research studies. Research studies have predominantly utilized single datasets, which may lack the size and diversity to represent the global spectrum of PM, thus limiting the models' generalizability. In classification tasks, the limited availability of imbalanced lesion distribution (e.g., “plus” lesions) in the META-PM classification of MM can result in overfitting. Second, lack of algorithm interpretability, particularly in tasks such as lesion localization (e.g., posterior staphyloma) and progression prediction, directly undermines clinicians' trust in AI outputs and their integration into practice. Third, deficiencies in clinical validation frameworks affect the spread and the use of AI for PM in clinical management. Of the included research studies above, overreliance on retrospective single-center data with limited real-world prospective validation limited prospective real-world validation research studies. The impact of variations in imaging equipment and quality remains understudied, despite its critical importance—especially given the heterogeneous image quality in primary care settings (e.g., county/township hospitals), which may limit the generalizability of AI models. Moreover, there is a need for a more in-depth exploration of model parameter tuning to enhance the accuracy of screening, grading, and prediction. While most algorithms struggle with detecting or localizing complications such as posterior staphyloma, some have shown promise in segmenting retinal lesion regions to improve detection rates. Last but not the least, the application of AI in PM and HM is still in the experimental stage and has not been widely put into clinical use, but some of the research studies mentioned above compared the accuracy of AI with the work of ophthalmic professionals, showing AI was better than or equal to the level of experts. Therefore, the development of AI and the advancement of its use in PM management is a major trend.

## 7 Conclusion

This review provides a comprehensive review of the recent advancements in using AI technologies to manage PM. It highlights the significant contributions of AI in improving screening and diagnostic processes, as well as in grading, classification, and predictive assessment. Integrating AI has notably enhanced the accuracy and efficiency of PM diagnosis and its complications, surpassing traditional methods. AI enables the segmentation and extraction of retinal lesions, which helps in understanding disease severity and predicting disease progression, crucial for its management. In summary, AI is revolutionizing PM clinical management by enhancing its intelligence, precision, and efficiency.

In summary, AI, through the application of DL and ML, has been effectively utilized in the domain of PM. Despite its demonstrated potential, several challenges remain. These include the need to address the limitations in processing low-quality images, refining disease progression models, and fostering interdisciplinary collaboration to advance research and application.

Looking ahead, the value of AI in the management of PM can be further enhanced by focusing on the following areas: (1) The development of more extensive and diverse multicenter PM datasets that include demographic parameters such as age, gender, and occupation, and increase the representation of rare findings such as “plus lesions”. (2) Researchers should concentrate on enhancing the image processing capabilities of AI models, particularly in improving clarity and noise reduction, while also considering the constraints of equipment in primary healthcare settings during clinical applications. (3) Although AI is not yet fully mature for predicting PM, future research can integrate predictive models with intervention strategies. For example, researchers can provide clinical interventions to patients at different risks and compare them with preclinical patients without interventions to see whether there is any difference in the patients' prognosis. By training AI models with substantial datasets and diverse cases, researchers can develop personalized prevention and treatment recommendations for PM patients based on their risk profiles.

## Data Availability

The original contributions presented in the study are included in the article/supplementary material, further inquiries can be directed to the corresponding author/s.

## References

[1] Ohno-Matsui K Wu PC Yamashiro K Vutipongsatorn K Fang Y Cheung CMG . IMI Pathologic Myopia. Invest Ophthalmol Vis Sci. (2021) 62:5. 10.1167/iovs.62.5.5 33909033 PMC8083114

[2] Morgan IG He M Rose KA. Epidemic of pathologic myopia. What can laboratory studies and epidemiology tell us? Retina. (2017) 37:989–97. 10.1097/IAE.0000000000001272 27617538

[3] Holden BA Fricke TR Wilson DA Jong M Naidoo KS Sankaridurg P . Global prevalence of myopia and high myopia and temporal trends from 2000 through 2050. Ophthalmology. (2016) 123:1036–42. 10.1016/j.ophtha.2016.01.006 26875007

[4] Morgan IG French AN Ashby RS Guo X Ding X He M . The epidemics of myopia: aetiology and prevention. Prog Retin Eye Res. (2018) 62:134–49. 10.1016/j.preteyeres.2017.09.004 28951126

[5] Wong TY Ferreira A Hughes R Carter G Mitchell P. Epidemiology and disease burden of pathologic myopia and myopic choroidal neovascularization: an evidence-based systematic review. Am J Ophthalmol. (2014) 157:9–25.e12. 10.1016/j.ajo.2013.08.010 24099276

[6] Liu H Li R Zhang Y Zhang K Yusufu M Liu Y . Economic evaluation of combined population-based screening for multiple blindness-causing eye diseases in China: a cost-effectiveness analysis. Lancet Global Health. (2023) 11:e456–e65. 10.1016/S2214-109X(22)00554-X 36702141

[7] Yang WH Shao Y Xu YW. Guidelines on clinical research evaluation of artificial intelligence in ophthalmology (2023). Int J Ophthalmol. (2023) 16:1361–72. 10.18240/ijo.2023.09.02 37724285 PMC10475621

[8] Ting DSW Pasquale LR Peng L Campbell JP Lee AY Raman R . Artificial intelligence and deep learning in ophthalmology. Br J Ophthalmol. (2019) 103:167–75. 10.1136/bjophthalmol-2018-313173 30361278 PMC6362807

[9] Li Y Yip MYT Ting DSW Ang M. Artificial intelligence and digital solutions for myopia. Taiwan J Ophthalmol. (2023) 13:142–50. 10.4103/tjo.TJO-D-23-00032 37484621 PMC10361438

[10] Hood DC La Bruna S Tsamis E Thakoor KA Rai A Leshno A . Detecting glaucoma with only OCT: Implications for the clinic, research, screening, and AI development. Prog Retin Eye Res. (2022) 90:101052. 10.1016/j.preteyeres.2022.101052 35216894

[11] Ting DSJ Foo VH Yang LWY Sia JT Ang M Lin H . Artificial intelligence for anterior segment diseases: emerging applications in ophthalmology. Br J Ophthalmol. (2021) 105:158–68. 10.1136/bjophthalmol-2019-315651 32532762

[12] Tan TE Wong TY. Diabetic retinopathy: looking forward to 2030. Front Endocrinol. (2022) 13:1077669. 10.3389/fendo.2022.1077669 36699020 PMC9868457

[13] Peng Z Ma R Zhang Y Yan M Lu J Cheng Q . Development and evaluation of multimodal AI for diagnosis and triage of ophthalmic diseases using ChatGPT and anterior segment images: protocol for a two-stage cross-sectional study. Front Artif Intell. (2023) 6:1323924. 10.3389/frai.2023.1323924 38145231 PMC10748413

[14] He HL Liu YX Song H Xu TZ Wong TY Jin ZB. Initiation of China Alliance of Research in High Myopia (CHARM): protocol for an AI-based multimodal high myopia research biobank. BMJ Open. (2023) 13:e076418. 10.1136/bmjopen-2023-076418 38151272 PMC10753734

[15] Cho BJ Shin JY Yu HG. Complications of pathologic myopia. Eye Contact Lens. (2016) 42:9–15. 10.1097/ICL.0000000000000223 26649982

[16] Sun G Wang X Xu L Li C Wang W Yi Z . Deep learning for the detection of multiple fundus diseases using ultra-widefield images. Ophthalmol Ther. (2023) 12:895–907. 10.1007/s40123-022-00627-3 36565376 PMC10011259

[17] Ludwig CA Moon J Garg I Miller JB. Ultra-Widefield Imaging for Evaluation of the Myopic Eye. Semin Ophthalmol. (2021) 36:185–90. 10.1080/08820538.2021.1887904 33620294

[18] Wang JD Liu MR Liu ML Zhang R Chen CX Cao K. An auxiliary diagnostic tool for common fundus diseases based on fundus color photography and light-weight classification models. Graefe's Arch Clin Exp Ophthalmol. (2024) 262:223–9. 10.1007/s00417-023-06182-2 37540261

[19] Zhu S Lu B Wang C Wu M Zheng B Jiang Q . Screening of common retinal diseases using six-category models based on EfficientNet. Front Med. (2022) 9:808402. 10.3389/fmed.2022.808402 35280876 PMC8904395

[20] Cao S Zhang R Jiang A Kuerban M Wumaier A Wu J . Application effect of an artificial intelligence-based fundus screening system: evaluation in a clinical setting and population screening. Biomed Eng Online. (2023) 22:38. 10.1186/s12938-023-01097-9 37095516 PMC10127070

[21] Gu C Wang Y Jiang Y Xu F Wang S Liu R . Application of artificial intelligence system for screening multiple fundus diseases in Chinese primary healthcare settings: a real-world, multicentre and cross-sectional study of 4795 cases. Br J Ophthalmol. (2024) 108:424–31. 10.1136/bjo-2022-322940 36878715 PMC10894824

[22] Lin PK Chiu YH Huang CJ Wang CY Pan ML Wang DW . PADAr: physician-oriented artificial intelligence-facilitating diagnosis aid for retinal diseases. J Med Imag. (2022) 9:044501. 10.1117/1.JMI.9.4.044501 35903415 PMC9311486

[23] Rauf N Gilani SO Waris A. Automatic detection of pathological myopia using machine learning. Sci Rep. (2021) 11:16570. 10.1038/s41598-021-95205-1 34400662 PMC8367943

[24] Cui J Zhang X Xiong F Chen CL. Pathological myopia image recognition strategy based on data augmentation and model fusion. J Healthc Eng. (2021) 2021:5549779. 10.1155/2021/5549779 34035883 PMC8118733

[25] Ren PF Tang XY Yu CY Zhu LL Yang WH Shen Y. Evaluation of a novel deep learning based screening system for pathologic myopia. Int J Ophthalmol. (2023) 16:1417–23. 10.18240/ijo.2023.09.07 37724265 PMC10475629

[26] Li M Liu S Wang Z Li X Yan Z Zhu R . MyopiaDETR: End-to-end pathological myopia detection based on transformer using 2D fundus images. Front Neurosci. (2023) 17:1130609. 10.3389/fnins.2023.1130609 36824210 PMC9941630

[27] Fang H Li F Wu J Fu H Sun X Orlando JI . Open fundus photograph dataset with pathologic myopia recognition and anatomical structure annotation. Scientific data. (2024) 11:99. 10.1038/s41597-024-02911-2 38245589 PMC10799845

[28] Wang R He J Chen Q Ye L Sun D Yin L . Efficacy of a deep learning system for screening myopic maculopathy based on color fundus photographs. Ophthalmol Ther. (2023) 12:469–84. 10.1007/s40123-022-00621-9 36495394 PMC9735275

[29] Tan TE Anees A Chen C Li S Xu X Li Z . Retinal photograph-based deep learning algorithms for myopia and a blockchain platform to facilitate artificial intelligence medical research: a retrospective multicohort study. Lancet Digital Health. (2021) 3:e317–e29. 10.1016/S2589-7500(21)00055-8 33890579

[30] Zhang W Zhao X Chen Y Zhong J Yi Z. DeepUWF: an automated ultra-wide-field fundus screening system *via* deep learning. IEEE J Biomed Health Inform. (2021) 25:2988–96. 10.1109/JBHI.2020.3046771 33361011

[31] Lu L Zhou E Yu W Chen B Ren P Lu Q . Development of deep learning-based detecting systems for pathologic myopia using retinal fundus images. Commun Biol. (2021) 4:1225. 10.1038/s42003-021-02758-y 34702997 PMC8548495

[32] Qian B Sheng B Chen H Wang X Li T Jin Y . A competition for the diagnosis of myopic maculopathy by artificial intelligence algorithms. JAMA Ophthalmol. (2024). 10.1001/jamaophthalmol.2024.3707 39325442 PMC11428027

[33] Li J Wang L Gao Y Liang Q Chen L Sun X . Automated detection of myopic maculopathy from color fundus photographs using deep convolutional neural networks. Eye Vision. (2022) 9:13. 10.1186/s40662-022-00285-3 35361278 PMC8973805

[34] Zhang J Xiao F Zou H Feng R He J. Self-supervised learning-enhanced deep learning method for identifying myopic maculopathy in high myopia patients. iScience. (2024) 27:110566. 10.1016/j.isci.2024.110566 39211543 PMC11359982

[35] Choi KJ Choi JE Roh HC Eun JS Kim JM Shin YK . Deep learning models for screening of high myopia using optical coherence tomography. Sci Rep. (2021) 11:21663. 10.1038/s41598-021-00622-x 34737335 PMC8568935

[36] Ye X Wang J Chen Y Lv Z He S Mao J . Automatic screening and identifying myopic maculopathy on optical coherence tomography images using deep learning. Transl Vis Sci Technol. (2021) 10:10. 10.1167/tvst.10.13.10 34751744 PMC8590175

[37] Li Y Feng W Zhao X Liu B Zhang Y Chi W . Development and validation of a deep learning system to screen vision-threatening conditions in high myopia using optical coherence tomography images. Br J Ophthalmol. (2022) 106:633–9. 10.1136/bjophthalmol-2020-317825 33355150 PMC9046742

[38] Ota-Itadani M Takahashi H Mao Z Igarashi-Yokoi T Yoshida T Ohno-Matsui K. Deep learning-based 3D OCT imaging for detection of lamina cribrosa defects in eyes with high myopia. Sci Rep. (2022) 12:22195. 10.1038/s41598-022-26520-4 36564438 PMC9789076

[39] Liu R Xuan M Wang DC Xiao O Guo XX Zhang J . Using choroidal thickness to detect myopic macular degeneration. Int J Ophthalmol. (2024) 17:317–23. 38371267 10.18240/ijo.2024.02.14 PMC10827620

[40] Zhao XJ Jiang HY Li YH Liu BQ Xu HX Zhou J . Correlations between the optic nerve head morphology and ocular biometrics in highly myopic eyes. Int J Ophthalmol. (2018) 11:997–1001. 29977814 10.18240/ijo.2018.06.17 PMC6010396

[41] Shi F Cheng XN Feng SL Yang CQ Diao SY Zhu WF . Group-wise context selection network for choroid segmentation in optical coherence tomography. Phys Med Biol. (2021) 66:245010. 10.1088/1361-6560/ac3a23 34787107

[42] Li M Zhou J Chen Q Zou H He J Zhu J . Choroid automatic segmentation and thickness quantification on swept-source optical coherence tomography images of highly myopic patients. Ann Transl Med. (2022) 10:620. 10.21037/atm-21-6736 35813325 PMC9263793

[43] Xu X Wang X Lin J Xiong H Wang M Tan H . Automatic segmentation and measurement of choroid layer in high myopia for OCT imaging using deep learning. J Digit Imaging. (2022) 35:1153–63. 10.1007/s10278-021-00571-x 35581408 PMC9582076

[44] Lin CY Chen HJ Chan YK Hsia WP Huang YL Chang CJ. Automatic fovea detection and choroid segmentation for choroidal thickness assessment in optical coherence tomography. Int J Ophthalmol. (2024) 17:1763–71. 10.18240/ijo.2024.10.01 39430032 PMC11422354

[45] Chen HJ Huang YL Tse SL Hsia WP Hsiao CH Wang Y . Application of artificial intelligence and deep learning for choroid segmentation in myopia. Transl Vis Sci Technol. (2022) 11:38. 10.1167/tvst.11.2.38 35212716 PMC8883159

[46] Wu W Gong Y Hao H Zhang J Su P Yan Q . Choroidal layer segmentation in OCT images by a boundary enhancement network. Front Cell Dev Biol. (2022) 10:1060241. 10.3389/fcell.2022.1060241 36438560 PMC9691264

[47] Wang Y Chen S Lin J Chen W Huang H Fan X . Vascular changes of the choroid and their correlations with visual acuity in pathological myopia. Invest Ophthalmol Vis Sci. (2022) 63:20. 10.1167/iovs.63.12.20 36378132 PMC9672896

[48] Luo H Sun J Chen L Ke D Zhong Z Cheng X . Compartmental analysis of three-dimensional choroidal vascularity and thickness of myopic eyes in young adults using SS-OCTA. Front Physiol. (2022) 13:916323. 10.3389/fphys.2022.916323 36160870 PMC9490056

[49] Xuan M Wang D Xiao O Guo X Zhang J Yin Q . Choroidal vascularity and axial length elongation in highly myopic children: a 2-year longitudinal investigation. Invest Ophthalmol Vis Sci. (2024) 65:7. 10.1167/iovs.65.10.7 39102263 PMC11309040

[50] Fujimoto S Miki A Maruyama K Mei S Mao Z Wang Z . Three-dimensional volume calculation of intrachoroidal cavitation using deep-learning-based noise reduction of optical coherence tomography. Transl Vis Sci Technol. (2022) 11:1. 10.1167/tvst.11.7.1 35802370 PMC9279919

[51] Cahyo DAY Wong DWK Yow AP Saw SM Schmetterer L. Volumetric choroidal segmentation using sequential deep learning approach in high myopia subjects. Annu Int Conf IEEE Eng Med Biol Soc. (2020) 2020:1286–9. 10.1109/EMBC44109.2020.9176184 33018223

[52] Mao J Deng X Ye Y Liu H Fang Y Zhang Z . Morphological characteristics of retinal vessels in eyes with high myopia: Ultra-wide field images analyzed by artificial intelligence using a transfer learning system. Front Med. (2022) 9:956179. 10.3389/fmed.2022.956179 36874950 PMC9982751

[53] Crincoli E Ferrara S Miere A Sacconi R Battista M Catania F . Correlation between AI-measured lacquer cracks extension and development of myopic choroidal neovascularization. Eye. (2023) 37:2963–8. 10.1038/s41433-023-02451-w 36859599 PMC10516917

[54] Wan C Wu J Li H Yan Z Wang C Jiang Q . Optimized-Unet: novel algorithm for parapapillary atrophy segmentation. Front Neurosci. (2021) 15:758887. 10.3389/fnins.2021.758887 34720868 PMC8550077

[55] He HL Liu YX Liu H Zhang X Song H Xu TZ . Deep learning-enabled vasculometry depicts phased lesion patterns in high myopia progression. Asia-Pacific J Ophthal. (2024) 13:100086. 10.1016/j.apjo.2024.100086 39053733

[56] Wan C Fang J Li K Zhang Q Zhang S Yang W . new segmentation algorithm for peripapillary atrophy and optic disk from ultra-widefield photographs(1). Comput Biol Med. (2024) 172:108281. 10.1016/j.compbiomed.2024.108281 38503096

[57] Xiao Y Zhao J Yu Y Ding X Liu S Bao W . SimpleCNN-UNet: an optic disc image segmentation network based on efficient small-kernel convolutions. Exp Syst Appl. (2024) 256:124935. 10.1016/j.eswa.2024.124935

[58] Chen L Zhou Y Gao S Li M Tan H Wan Z. ARA-net: an attention-aware retinal atrophy segmentation network coping with fundus images. Front Neurosci. (2023) 17:1174937. 10.3389/fnins.2023.1174937 37179557 PMC10174230

[59] Zhang Z Xu Y Liu J Wong DW Kwoh CK Saw SM . Automatic diagnosis of pathological myopia from heterogeneous biomedical data. PLoS ONE. (2013) 8:e65736. 10.1371/journal.pone.0065736 23799040 PMC3683061

[60] Zhang R Dong L Yang Q Zhou W Wu H Li Y . Screening for novel risk factors related to high myopia using machine learning. BMC Ophthalmol. (2022) 22:405. 10.1186/s12886-022-02627-0 36229775 PMC9558412

[61] Fang Z Xu Z He X Han W. Artificial intelligence-based pathologic myopia identification system in the ophthalmology residency training program. Front Cell Dev Biol. (2022) 10:1053079. 10.3389/fcell.2022.1053079 36407106 PMC9669055

[62] Wan C Li H Cao GF Jiang Q Yang WH. An artificial intelligent risk classification method of high myopia based on fundus images. J Clin Med. (2021) 10:4488. 10.3390/jcm10194488 34640509 PMC8509666

[63] Huang X He S Wang J Yang S Wang Y Ye X. Lesion detection with fine-grained image categorization for myopic traction maculopathy (MTM) using optical coherence tomography. Med Phys. (2023) 50:5398–409. 10.1002/mp.16623 37490302

[64] Hemelings R Elen B Blaschko MB Jacob J Stalmans I De Boever P. Pathological myopia classification with simultaneous lesion segmentation using deep learning. Comput Methods Programs Biomed. (2021) 199:105920. 10.1016/j.cmpb.2020.105920 33412285

[65] Du R Xie S Fang Y Igarashi-Yokoi T Moriyama M Ogata S . Deep learning approach for automated detection of myopic maculopathy and pathologic myopia in fundus images. Ophthalmology Retina. (2021) 5:1235–44. 10.1016/j.oret.2021.02.006 33610832

[66] Han YX Guo XX Wang YX Jonas JB Chen X Wang XF. Automated posterior scleral topography assessment for enhanced staphyloma visualization and quantification with improved maculopathy correlation. Transl Vis Sci Technol. (2024) 13:41. 10.1167/tvst.13.10.41 39476086 PMC11534019

[67] Zheng B Zhang M Zhu S Wu M Chen L Zhang S . Research on an artificial intelligence-based myopic maculopathy grading method using EfficientNet. Indian J Ophthalmol. (2024) 72:S53–s9. 10.4103/IJO.IJO_48_23 38131543 PMC10833160

[68] Sogawa T Tabuchi H Nagasato D Masumoto H Ikuno Y Ohsugi H . Accuracy of a deep convolutional neural network in the detection of myopic macular diseases using swept-source optical coherence tomography. PLoS ONE. (2020) 15:e0227240. 10.1371/journal.pone.0227240 32298265 PMC7161961

[69] Du Y Chen Q Fan Y Zhu J He J Zou H . Automatic identification of myopic maculopathy related imaging features in optic disc region *via* machine learning methods. J Transl Med. (2021) 19:167. 10.1186/s12967-021-02818-1 33902640 PMC8074495

[70] He HL Liu YX Chen XY Ling SG Qi Y Xiong Y . Fundus tessellated density of pathologic myopia. Asia-Pacific J Ophthalmol. (2023) 12:604–13. 10.1097/APO.0000000000000642 38079255

[71] Gong W Wang J Deng J Chen J Zhu Z Seth I . Quantification of fundus tessellation reflects early myopic maculopathy in a large-scale population of children and adolescents. Transl Vis Sci Technol. (2024) 13:22. 10.1167/tvst.13.6.22 38922627 PMC11216261

[72] Sun Y Li Y Zhang F Zhao H Liu H Wang N . A deep network using coarse clinical prior for myopic maculopathy grading. Comput Biol Med. (2023) 154:106556. 10.1016/j.compbiomed.2023.106556 36682177

[73] Shao L Zhang QL Long TF Dong L Zhang C Da Zhou W . Quantitative assessment of fundus tessellated density and associated factors in fundus images using artificial intelligence. Transl Vis Sci Technol. (2021) 10:23. 10.1167/tvst.10.9.23 34406340 PMC8383900

[74] Lu L Ren P Tang X Yang M Yuan M Yu W . AI-Model for Identifying Pathologic Myopia Based on Deep Learning Algorithms of Myopic Maculopathy Classification and “Plus” Lesion Detection in Fundus Images. Front Cell Dev Biol. (2021) 9:719262. 10.3389/fcell.2021.719262 34722502 PMC8554089

[75] Niu YN He HL Chen XY Ling SG Dong Z Xiong Y . A novel grading system for diffuse chorioretinal atrophy in pathologic myopia. Ophthalmol Ther. (2024) 13:1171–84. 10.1007/s40123-024-00908-z 38441856 PMC11039581

[76] Li Y Li H Rui X Wang Y Zhu S Huang M . Choroidal vascular changes in early-stage myopic maculopathy from deep learning choroidal analysis: a hospital-based SS-OCT study. Eye Vision. (2024) 11:32. 10.1186/s40662-024-00398-x 39107859 PMC11301841

[77] Wu Z Cai W Xie H Chen S Wang Y Lei B . Predicting optical coherence tomography-derived high myopia grades from fundus photographs using deep learning. Front Med. (2022) 9:842680. 10.3389/fmed.2022.842680 35308524 PMC8927672

[78] He X Ren P Lu L Tang X Wang J Yang Z . Development of a deep learning algorithm for myopic maculopathy classification based on OCT images using transfer learning. Front Publ Health. (2022) 10:1005700. 10.3389/fpubh.2022.1005700 36211704 PMC9532624

[79] Zhou Y Chen X Li TY Lin SQ Sheng B Liu RH . GAMNet: a gated attention mechanism network for grading myopic traction maculopathy in OCT images. Visual Computer. (2025) 41:1097–108. 10.1007/s00371-024-03386-3

[80] Zhang Z Gao Q Fang D Mijit A Chen L Li W . Effective automatic classification methods *via* deep learning for myopic maculopathy. Front Med. (2024) 11:1492808. 10.3389/fmed.2024.1492808 39606624 PMC11598530

[81] Wan C Fang J Hua X Chen L Zhang S Yang W. Automated detection of myopic maculopathy using five-category models based on vision outlooker for visual recognition. Front Comput Neurosci. (2023) 17:1169464. 10.3389/fncom.2023.1169464 37152298 PMC10157024

[82] Zhu SJ Zhan HD Wu MN Zheng B Liu BQ Zhang SC . Research on classification method of high myopic maculopathy based on retinal fundus images and optimized ALFA-Mix active learning algorithm. Int J Ophthalmol. (2023) 16:995–1004. 10.18240/ijo.2023.07.01 37465510 PMC10333255

[83] Tang J Yuan M Tian K Wang Y Wang D Yang J . An artificial-intelligence-based automated grading and lesions segmentation system for myopic maculopathy based on color fundus photographs. Transl Vis Sci Technol. (2022) 11:16. 10.1167/tvst.11.6.16 35704327 PMC9206390

[84] Meng J Song Y He W Lu ZL Chen Y Wei L . A novel artificial intelligence-based classification of highly myopic eyes based on visual function and fundus features. Transl Vis Sci Technol. (2024) 13:12. 10.1167/tvst.13.9.12 39235401 PMC11379094

[85] Zhao J Yu Y Li Y Li F Zhang Z Jian W . Development and validation of predictive models for myopia onset and progression using extensive 15-year refractive data in children and adolescents. J Transl Med. (2024) 22:289. 10.1186/s12967-024-05075-0 38494492 PMC10946190

[86] Yoo TK Ryu IH Kim JK Lee IS. Deep learning for predicting uncorrected refractive error using posterior segment optical coherence tomography images. Eye. (2022) 36:1959–65. 10.1038/s41433-021-01795-5 34611313 PMC9500028

[87] Wang Y Du R Xie S Chen C Lu H Xiong J . Machine learning models for predicting long-term visual acuity in highly myopic eyes. JAMA Ophthalmol. (2023) 141:1117–24. 10.1001/jamaophthalmol.2023.4786 37883115 PMC10603576

[88] Chen Y Yang S Liu R Xiong R Wang Y Li C . Forecasting myopic maculopathy risk over a decade: development and validation of an interpretable machine learning algorithm. Invest Ophthalmol Vis Sci. (2024) 65:40. 10.1167/iovs.65.6.40 38935031 PMC11216278

[89] Kim YC Chang DJ Park SJ Choi IY Gong YS Kim HA . Machine learning prediction of pathologic myopia using tomographic elevation of the posterior sclera. Sci Rep. (2021) 11:6950. 10.1038/s41598-021-85699-0 33772040 PMC7997908

[90] Lu HC Chen HY Huang CJ Chu PH Wu LS Tsai CY. Predicting axial length from choroidal thickness on optical coherence tomography images with machine learning based algorithms. Front Med. (2022) 9:850284. 10.3389/fmed.2022.850284 35836947 PMC9273745

[91] Li S Li M Wu J Li Y Han J Cao W . Development and validation of a routine blood parameters-based model for screening the occurrence of retinal detachment in high myopia in the context of PPPM. EPMA J. (2023) 14:219–33. 10.1007/s13167-023-00319-3 37275550 PMC10015135

[92] Shi XH Dong L Zhang RH Wei WB. Association between weight-adjusted waist index and myopia in adolescents and young adults: results from NHANES 1999-2008. BMC Ophthalmol. (2024) 24:14. 10.1186/s12886-024-03282-3 38191303 PMC10775622

[93] Lin H Long E Ding X Diao H Chen Z Liu R . Prediction of myopia development among Chinese school-aged children using refraction data from electronic medical records: A retrospective, multicentre machine learning study. PLoS Med. (2018) 15:e1002674. 10.1371/journal.pmed.1002674 30399150 PMC6219762

[94] Foo LL Lim GYS Lanca C Wong CW Hoang QV Zhang XJ . Deep learning system to predict the 5-year risk of high myopia using fundus imaging in children. NPJ Digit Med. (2023) 6:10. 10.1038/s41746-023-00752-8 36702878 PMC9879938

[95] Li J Zeng S Li Z Xu J Sun Z Zhao J . Accurate prediction of myopic progression and high myopia by machine learning. Precis Clin Med. (2024) 7:pbae005. 10.1093/pcmedi/pbae005 38558949 PMC10981449

[96] Guan J Zhu Y Hu Q Ma S Mu J Li Z . Prevalence patterns and onset prediction of high myopia for children and adolescents in southern china *via* real-world screening data: retrospective school-based study. J Med Internet Res. (2023) 25:e39507. 10.2196/39507 36857115 PMC10018376

[97] Clark R Lee SS Du R Wang Y Kneepkens SCM Charng J . A new polygenic score for refractive error improves detection of children at risk of high myopia but not the prediction of those at risk of myopic macular degeneration. EBioMedicine. (2023) 91:104551. 10.1016/j.ebiom.2023.104551 37055258 PMC10203044

[98] Liu F Yu XH Wang YC Cao M Xie LF Liu J . Quantitative analysis of optic disc changes in school-age children with ametropia based on artificial intelligence. Int J Ophthalmol. (2023) 16:1727–33. 10.18240/ijo.2023.11.01 38028515 PMC10626368

[99] Kang EY Yeung L Lee YL Wu CH Peng SY Chen YP . A multimodal imaging-based deep learning model for detecting treatment-requiring retinal vascular diseases: model development and validation study. JMIR Med Inform. (2021) 9:e28868. 10.2196/28868 34057419 PMC8204240

[100] Zhao Y Zhao Z Yang J Li L Nasseri MA Zapp D. AI-based fully automatic analysis of retinal vascular morphology in pediatric high myopia. BMC Ophthalmol. (2024) 24:415. 10.1186/s12886-024-03682-5 39334037 PMC11437631

[101] Liu L Zhong L Zeng L Liu F Yu X Xie L . Quantitative analysis of retinal vascular parameters changes in school-age children with refractive error using artificial intelligence. Front Med. (2024) 11:1528772. 10.3389/fmed.2024.1528772 39811156 PMC11729343

[102] Zhou Y Dai M Sun L Tang X Zhou L Tang Z . The accuracy of intraocular lens power calculation formulas based on artificial intelligence in highly myopic eyes: a systematic review and network meta-analysis. Front Publ Health. (2023) 11:1279718. 10.3389/fpubh.2023.1279718 38026369 PMC10670805

[103] Guo D He W Wei L Song Y Qi J Yao Y . The Zhu-Lu formula: a machine learning-based intraocular lens power calculation formula for highly myopic eyes. Eye Vision. (2023) 10:26. 10.1186/s40662-023-00342-5 37259154 PMC10233923

[104] Wei L He W Wang J Zhang K Du Y Qi J . An optical coherence tomography-based deep learning algorithm for visual acuity prediction of highly myopic eyes after cataract surgery. Front Cell Dev Biol. (2021) 9:652848. 10.3389/fcell.2021.652848 34124042 PMC8187805

[105] Yang M Han J Park JI Hwang JS Han JM Yoon J . Prediction of visual acuity in pathologic myopia with myopic choroidal neovascularization treated with anti-vascular endothelial growth factor using a deep neural network based on optical coherence tomography images. Biomedicines. (2023) 11:2238. 10.3390/biomedicines11082238 37626734 PMC10452208

[106] Sawai Y Miyata M Uji A Ooto S Tamura H Ueda-Arakawa N . Usefulness of denoising process to depict myopic choroidal neovascularisation using a single optical coherence tomography angiography image. Sci Rep. (2020) 10:6172. 10.1038/s41598-020-62607-6 32277172 PMC7148361

[107] Cheng J Li Z Gu Z Fu H Wong DWK Liu J. Structure-preserving guided retinal image filtering and its application for optic disk analysis. IEEE Trans Med Imaging. (2018) 37:2536–46. 10.1109/TMI.2018.2838550 29994522

